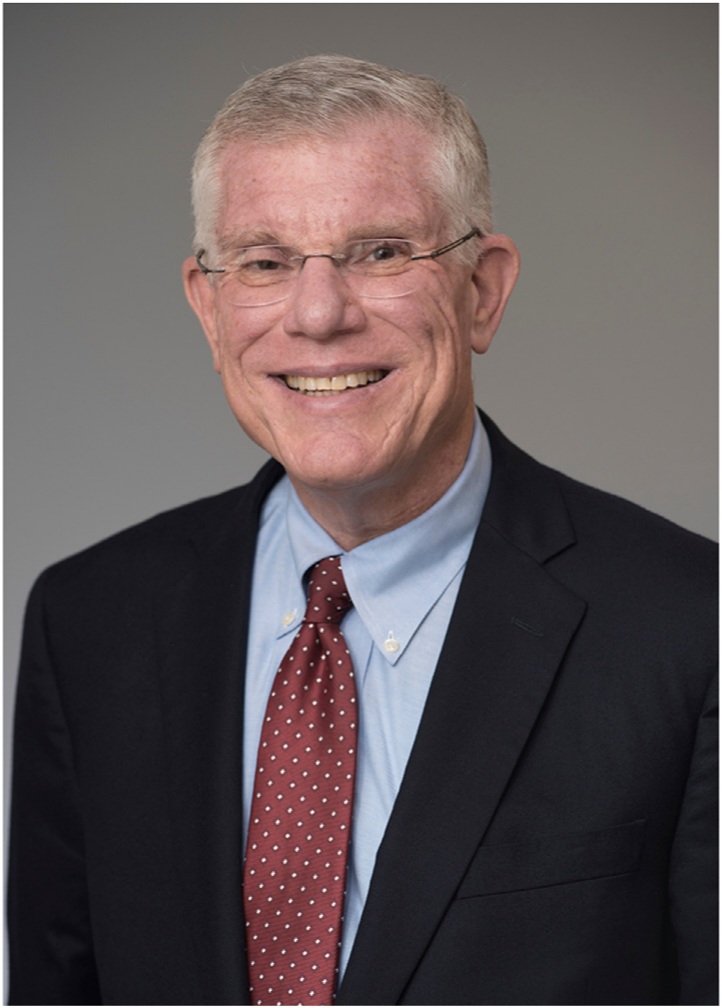# Iron Overload in Non-HFE Liver Disease: Not all Iron is Ready to Strike

**DOI:** 10.1016/j.gastha.2023.10.004

**Published:** 2023-10-13

**Authors:** Paul Martin, Lawrence S. Friedman

**Affiliations:** 1 Miller School of Medicine, University of Miami, Miami, Florida; 2 Department of Medicine, Newton-Wellesley Hospital, Newton, Massachusetts; 3 Massachusetts General Hospital, Harvard Medical School, and Tufts University School of Medicine, Boston, Massachusetts

Pathological iron overload with end-organ damage in hemochromatosis occurs in individuals who are homozygous for the major mutation, C282Y. Phenotypic hemochromatosis occurs much less frequently in compound heterozygotes with one C282Y mutation and one H63D mutation. Iron overload can be confirmed by magnetic resonance imaging, which shows a loss of signal intensity in affected tissues and avoids the need for liver biopsy.

The serum ferritin level, an acute phase reactant, may be elevated for reasons other than iron overload, including infection and malignancy; in such cases, the iron saturation is usually normal. In patients with liver disease, iron overload is not restricted to patients with genetic hemochromatosis. In nonalcoholic fatty liver disease (NAFLD), up to one-third of patients have an elevated iron saturation (> 45%) and an elevated serum ferritin level. Iron accumulation in NAFLD can occur in hepatocytes, the reticuloendothelial system, or both. Deposition of iron in the reticuloendothelial system has been implicated in more severe liver disease (steatohepatitis and fibrosis) in NAFLD. Hepatic iron accumulation is also frequent in alcohol-associated liver disease. In chronic hepatitis B and C, accumulation of hepatic iron is also recognized. In any patient with chronic liver disease, an elevated serum ferritin or an elevated iron saturation should prompt testing for HFE mutations to exclude hemochromatosis.

**Figure:**
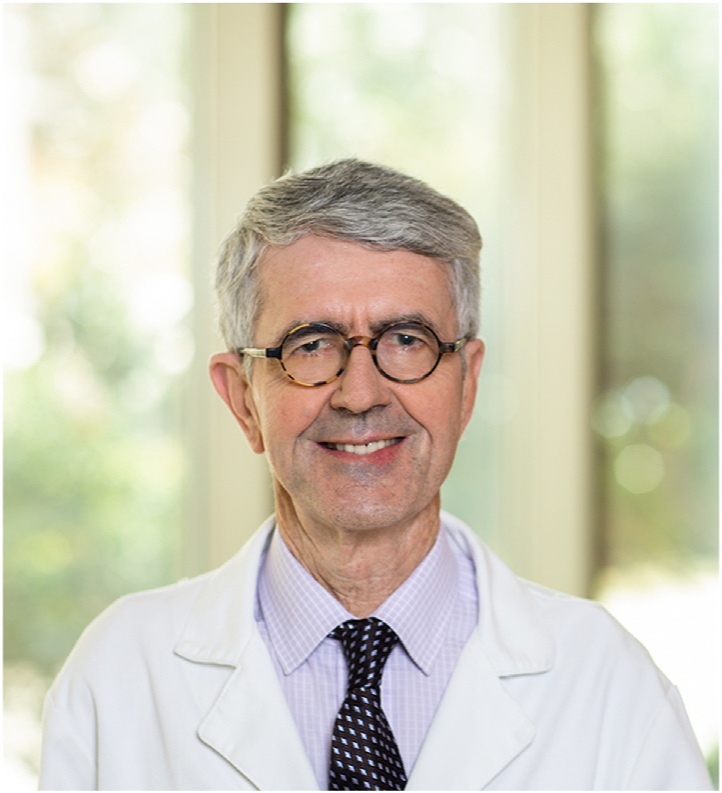

**Figure:**